# An Explanatory Model for the Relationship between Motivation in Sport, Victimization, and Video Game Use in Schoolchildren

**DOI:** 10.3390/ijerph15091866

**Published:** 2018-08-29

**Authors:** Manuel Castro-Sánchez, Ramón Chacón-Cuberos, José Luis Ubago-Jiménez, Edson Zafra-Santos, Félix Zurita-Ortega

**Affiliations:** 1 Department of Education, University of Almería, 04120 Almería, Spain; mcastros@ual.es; 2 Department of Integrated Didactics, University of Huelva, 21007 Huelva, Spain; 3 Department of Didactics of Musical, Plastic and Corporal Expression, University of Granada, 18071 Granada, Spain; jlubago@ugr.es (J.L.U.-J.); felixzo@ugr.es (F.Z.-O.); 4 Kinesiology School, University Santo Tomas, 837003 Santiago, Chile; ezafra@santotomas.cl

**Keywords:** motivational climate in sport, physical activity, bullying, problematic use of video games, children

## Abstract

(1) Background: Society is changing amazingly fast, and this is bringing about changes in the way that people spend their free time. In the 21st century, free time is increasingly spent using technological devices such as video games, thus increasing levels of sedentariness. The aim of the present study was to define an explanatory model for the problematic use of video games, physical activity, motivational climate in sports, and victimization in schoolchildren, and to analyze the relationships between these variables according to gender; (2) Methods: A total of 734 schoolchildren, of both sexes, participated in this research study. They were aged from 10 to 12 and lived in the province of Granada (Spain). The main instruments used were the questionnaires PMCSQ-2, PAQ-C, QERV, and SVS. A multigroup structural equation model was used, which had an excellent fit (χ^2^ = 319.472; df = 72; *p* < 0.001; CFI = 0.962; NFI = 0.952; IFI = 0.962; RMSEA = 0.048); (3) Results: The practice of physical activity was related negatively and indirectly to the problematic use of video games ((r = −0.085, boys); (r = −0.081, girls)), and this in turn was related positively and directly to victimization ((r = 0.094, boys); (r = 0.174, girls)). Additionally, task climate was inversely related to the problematic use of video games for girls (r = −0.133), and ego climate was directly related to the use of these devices only with regard to boys (r = 0.250). (4) Conclusions: It must be noted that schoolchildren’s pathological use of video games is closely related to lower levels of physical activity. In addition, those motivational climates in sports that are oriented towards performance exacerbate this pathological behavior, which accentuates the importance of promoting motivational climates that are oriented towards tasks in schoolchildren.

## 1. Introduction

Society is changing at an unprecedented rate, which is affecting the way in which people spend their free time. The youngest in society were born in the middle of the “technological era”, their growing up coinciding with an unstoppable surge in technological advancement and the development of electronic entertainment devices never seen before [1,2]. Therefore, in the 21st century, society is facing the problem of the pathological use of digital entertainment devices, which is especially prevalent in the youngest sectors of the population, such as schoolchildren [3].

Authors such as Chacón-Cuberos et al. [4] or Verheijen et al. [5], indicate that parents have little control over their children’s use of this kind of technology, due to its rapid spread throughout society. The problematic use of video games in schoolchildren is also a risk factor for diverse health problems at the cognitive, social, and physical levels [6,7,8]. A great deal of research has analyzed the negative consequences of the problematic use of video games. Some of these are physical, such as hormonal changes and various eye problems; some are social, such as the deterioration and loss of socio-emotional skills; and others are cognitive, such as increasing anxiety, which can lead to depression in serious cases [9,10]. In this regard, a great deal of research has analyzed the negative behavioral consequences that stem from the abuse of this kind of technology, revealing a reduction in physical activity that leads to a rise in sedentary lifestyles and obesity [11], all of which are associated with poorer health in general [12]. Another of the negative consequences of video games abuse is increased aggressiveness, a direct link having been identified between these two factors [13,14]. This is attributed to the violent nature of many video games, whose use is considered a risk factor for violent behavior [15,16].

In this sense, schoolchildren are very easily influenced and readily modify their behavior patterns. Video games are seen as the ultimate audiovisual medium, but they disseminate imitable negative behaviors that adversely affect anyone who has not undergone an appropriate socialization process [17,18]. By playing video games with a high content of aggressiveness, young people come to associate violence with positive emotions such as the enjoyment the game provides, which results in the young person becoming desensitized to such violent behavior [19,20]. For that reason, as indicated by Ditrrick et al. [21] and Kimmig et al. [22], constant use of these kind of video games by young people causes a fall in their levels of anxiety and fear in the face of violent situations, which in some cases causes them to lose their ability to feel empathy for victims and to identify instead with the aggressors, which in turn may bring about a greater tolerance of aggression and even engender violent behavior towards peers.

According to this basis, recent studies such as those by Boxer et al. [23] or DeCamp & Ferguson [24] connect the abuse of screen-based entertainment such as video games and TV with an increase in victimization, a deterioration of academic performance, and the proliferation of symptoms related to depression that can lead to intrafamilial violence. In the face of all this, physical activity becomes a fundamental tool for counteracting the spread of unhealthy habits induced by greater sedentariness related to the use of video games. It can also prevent addiction problems; perhaps even the use of harmful substances such as alcohol, tobacco and other drugs [25,26,27].

For these reasons, studying the process of motivating schoolchildren to take up physical activity is considered essential, given that this is ultimately what will determine whether they continue with sporting activities, or either abandon them or replace them with sedentary habits related to the use of digital entertainment and the consumption of harmful substances [28]. The study of motivation towards sport is vital since, depending on the type of sport practiced, it can act both as a risk factor and as a factor preventing the adoption of unhealthy behaviors [29,30]. With regard to the study of motivation, in the context of physical activity and sport, the Achievement Goal Theory constitutes one of the most popular theoretical frameworks. This theory is based on the fundamental idea that motivation towards the practice of physical activity depends on the objectives that the person wants to achieve, which are known as achievement goals [31,32].

On the basis of their work on motivational orientations, authors such as Lochbaum et al. [33] indicate the existence of two different motivational climates: one is oriented towards the task and is characterized by more self-determined or intrinsic motivations, where the person practices physical activity for pleasure or fun, which promotes greater adherence to the practice of sport; the other is oriented towards ego and is characterized by a less self-determined or extrinsic type of motivation, usually associated with competition and external rewards, and thus promoting rivalry between peers because of their fear of failure, which is associated more with the abandonment of physical activity [34,35]. In this line, ego-oriented climates could be related to non-adaptive behaviors such as aggressiveness and the problematic use of video games, while task-oriented climates could be associated with adaptive behaviors and healthy habits.

Therefore, the present research study has the following objectives: (a) to define and contrast an explanatory model for the motivational climate, the practice of physical activity, the use of video games, and victimization among schoolchildren, and (b) to analyze the relationships between motivational climate, the practice of physical activity, the use of video games, and victimization by gender (boys/girls), by the means of a multigroup structural equation analysis.

## 2. Materials and Methods

### 2.1. Subjects and Design

This descriptive and cross-sectional study was conducted on a sample of 734 schoolchildren, both boys and girls (45.2% boys and 54.8% girls), aged between 10 and 12 (M = 10.88; SD = 0.69), in fifth and sixth primary grade in the city of Granada. The sample was chosen by convenience sampling, with the requirement being that the participants be at the third stage of primary education. The children in the sample were from 11 eleven different schools in the city of Granada, and all the schools that agreed to participate were asked to do so on a voluntary basis. It should be pointed out that in order to avoid data duplication, the non-repetition of subjects was guaranteed by individualized monitoring during data collection.

### 2.2. Measures

Problematic use of video games: the instrument CERV (Questionnaire of Experiences Related to Video Games) originally developed by Chamarro et al. [36] was used. This instrument, which was validated on a sample of 7168 Spanish adolescents by Chamarro et al. [36], is made up of 17 negatively formulated items, graded by the means of a Likert scale with four options (1 = Never; 2 = Sometimes; 3 = Often; 4 = Always). The items are totaled up to describe the person’s behavior in relation to the use of video games, classifying them in tertiles to categorize the variables into: “No problems”, “Potential problems” and “Serious problems”. The reliability of the original instrument by Chamarro et al. [36] was α = 0.87, and in the present study it is α = 0.91.

Practice of physical activity: the questionnaire “Physical Activity Questionnaire for older Children (PAQ-C)” was used in its validated and adapted version in Spanish [37]. The questionnaire assesses moderate to vigorous physical activity practiced during the last seven days, by the means of 10 questions about the type and frequency of the activities carried out. The scores for each question ranged from 1 to 5, and calculation of the final score indicated a higher or lower frequency of the practice of physical activity. This instrument achieved a consistency of α = 0.77, which was acceptable and very similar to the value obtained by Kowalski et al. [37] in the original study (α = 0.79).

Motivational climate in sport (PMCSQ-2). This questionnaire derives from the original version by Newton et al. [38], adapted to Spanish by González-Cutre et al. [39]. It is made up of 33 items graded by means of a Likert scale with five options ranging from 1 = Totally Disagree to 5 = Totally Agree. The questionnaire establishes two categories: Task Climate, with its categories Cooperative Learning, Effort/Improvement and Important Role, and Ego Climate, with its categories Punishment for Mistakes, Unequal Recognition and Team Member Rivalry. The internal consistency (Cronbach’s alpha) of the instrument obtained by González-Cutre et al. [39] in its Spanish version was α = 0.90 for Ego Climate (α = 0.77 for punishment for mistakes, α = 0.87 for unequal recognition, α = 0.61 for team member rivalry) and α = 0.84 for Task Climate (α = 0.65 for cooperative learning, α = 0.70 for effort/improvement, α = 0.70 for important role). The present research study obtained α = 0.89 for Ego Climate (α = 0.92 for punishment for mistakes, α = 0.91 for unequal recognition, α = 0.68 for member rivalry) and α = 0.93 for Task Climate (α = 0.83 for cooperative learning, α = 0.84 for effort/improvement, α = 0.86 for important role).

School Victimization Scale: This questionnaire was created by Mynard & Joseph [40] and adapted to Spanish by Cava et al. [41]. It is made up of 20 items graded by the means of a Likert scale (1 = Never; 4 = Always), addressing three types of victimization; Physical Victimization, Verbal Victimization, and Relational Victimization. In the original study, Mynard & Joseph [40] obtained an internal consistency (Cronbach’s alpha) of α = 0.77. In this study, the coefficient was higher, at α = 0.93 (α = 0.88 for Relational Victimization; α = 0.86 for Physical Victimization; α = 0.84 for Verbal Victimization).

### 2.3. Procedure

First, through the Faculty of Education of the University of Granada, with the cooperation of the Department of Education of the regional government of Andalucia, a request for collaboration was made to the participating schools in the city of Granada, which had been selected by convenience sampling. These schools were informed of the nature of the research study, and collaboration of the students was requested. Secondly, a consent form was sent to the legal guardians of the teenagers requesting their informed consent, since the participants were minors.

The anonymity of the data collected was guaranteed throughout the process, it being made clear that the information would be used for scientific purposes only. The research team was constantly available in order to solve any doubts. The questionnaires were all completed without any problems or abnormalities to report. Finally, teachers, school counselors, and responsible staff were thanked for their collaboration and told that they would soon be sent a report on the data obtained, with confidentiality preserved at all times.

After data collection, a total of 52 questionnaires had to be rejected because they were not correctly completed. The present research study followed the guidelines set out in the Declaration of Helsinki (World Medical Association, 2008) for research projects, as well as the national legislation regulating clinical drug trials (Royal Decree 223/2004 of 6 February), biomedical research (Law 14/2007 of 3 July), and the protection of personal data (Organic Law 15/1999 of 13 December).

### 2.4. Data Analysis

The statistical software used was IBM SPSS^®^ version 22.0 (IBM Corp, Armonk, NY, USA) for Windows, for the analysis of basic descriptors. The program IBM AMOS^®^ 23 (IBM Corp, Armonk, NY, USA) was used for analysis of the relationships between constructs in the structural model. Once the theoretical model had been developed, a path analysis was carried out, considering matrix relationships derived from a multigroup analysis classifying participants by gender as a grouping variable. Therefore, two different structural models were established in order to check whether relationships between variables vary according to gender.

**Figure 1:**
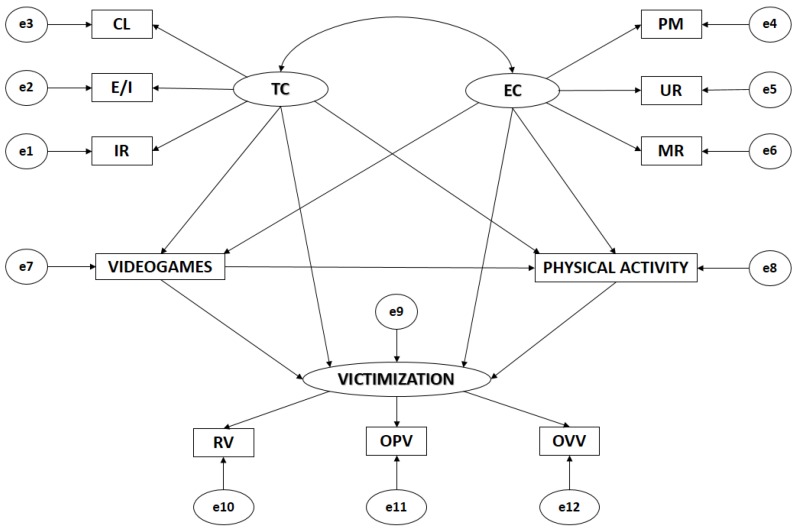
Model theories. Note1: TC, Task Climate; CL, Cooperative Learning; E/I, Effort/Improvement; IR, Important Role; EC, Ego Climate; MR, Member Rivalry; PM, Punishment for Mistakes; UR, Unequal Recognition; VIDEO GAMES, Use of video games; PA, Physical Activity; VICTIMIZATION, Victimization; RV, Relational Victimization; OPV, Overt Physical Victimization; OVV, Overt Verbal Victimization.

Path routes are created using 11 observable variables and three latent variables in order to determine indicators (Figure 1). These indicators give causal explanations of the latent variables by observing relationships between the indicators, considering the reliability of the measurements. Additionally, measurement errors are included in the observable variables in order to control them directly. One-way arrows are influence lines between latent and observable variables, and they are interpreted as multivariate regression coefficients. Two-way arrows show the relationship between latent variables, also representing the regression coefficients.

Motivational climate oriented towards task (TC) and motivational climate oriented towards ego (EC) act as exogenous variables, and each of them is inferred by three indicators. For task climate, the indicators are IR (Important Role), E/I (Effort/Improvement), and CL (Cooperative Learning). For ego climate, they are PM (Punishment for Mistakes), UR (Unequal Recognition), and MR (Team Member Rivalry). The use of video games (VIDEO GAMES) acts as an endogenous variable, receiving the effect of task climate (TC) and ego climate (EC). The practice of physical activity (PHYSICAL ACTIVITY) also acts as an endogenous variable, receiving the effect of task climate (TC), ego climate (EC), and the use of video games (VIDEO GAMES). Likewise, victimization (VICTIMIZATION) acts as an endogenous variable, receiving the effect of the use of video games (VIDEO GAMES), the practice of physical activity (PHYSICAL ACTIVITY), task climate (TC), and ego climate (EC).

The model fit was checked in order to verify its compatibility and the empirical information obtained. Fit reliability was based on the fit indexes established by Marsh [42]. In relation to Chi-square, non-significant values associated with p denote a good model fit. The Comparative Fit Index (CFI) will be acceptable if it is higher than 0.90, and excellent if it is higher than 0.95. The Normed Fit Index (NFI) will be acceptable if it is higher than 0.90. The Incremental Fit Index (IFI) will be acceptable if it is higher than 0.90, and excellent if it is higher than 0.95. Finally, the Root Mean Square Error of Approximation (RMSEA) will be excellent if it is lower than 0.05 and acceptable if it is lower than 0.08.

## 3. Results

The proposed structural equation model according to schoolchildren’s gender obtained a good fit in all assessment indices. The Chi-square had a significant value of p (χ^2^ = 319,472; DF = 72; *p* < 0.001). Nevertheless, this index cannot be interpreted in a standardized way, which poses an additional problem by its sensitivity to sample size [42]. Therefore, other standardized fit indices that were less sensitive to sample size were used. The comparative fit index (CFI) had a value of 0.962, which was excellent. The normed fit index (NFI) had a value of 0.952, and the incremental fit index (IFI) a value of 0.962, both also being acceptable. The root mean square error of approximation (RMSEA) had an excellent value of 0.048.

**Figure 2:**
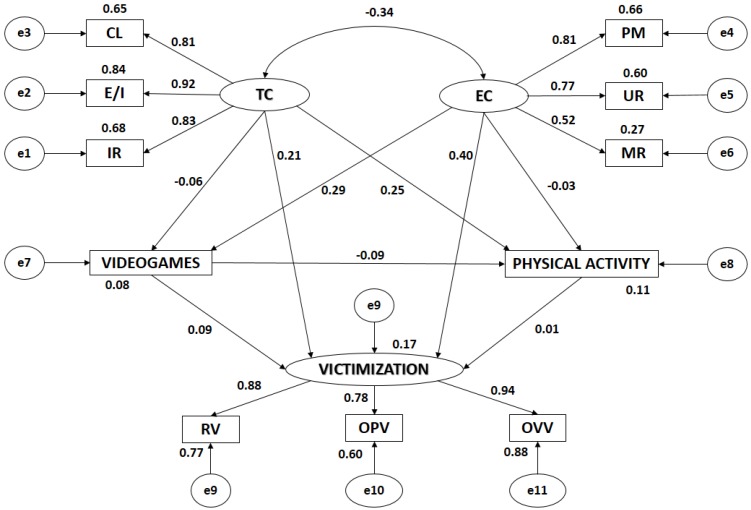
Structural equation model for boys. Note: TC, Task Climate; CL, Cooperative Learning; E/I, Effort/Improvement; IR, Important Role; EC, Ego Climate; MR, Member Rivalry; PM, Punishment for Mistakes; UR, Unequal Recognition; VIDEO GAMES, Use of video games; PA, Physical Activity; VICTIMIZATION, Victimization; RV, Relational Victimization; OPV, Overt Physical Victimization; OVV, Overt Verbal Victimization.

Figure 2 and Table 1 show the estimated values of the parameters in the structural model for school boys. These must be of a suitable magnitude, and the effects must be significantly different from zero. No improper estimations such as negative variances should be found.

Statistically significant relations were found at the *p* < 0.005 level in all associations between motivational climate and its dimensions, all of these being positive and direct. The relationship between Task Climate and Ego Climate was significant at the *p* < 0.005 level, being negative and indirect (r = −0.339). Likewise, statistically significant relations were found at the *p* < 0.005 level in all associations between Victimization and its indicators, all of these being positive and direct.

Analyzing the influence of the indicators in the latent variables, it could be seen that all of them had statistically significant differences at the *p* < 0.005 level, all of these being positive and direct. In Task Climate, the indicator with the highest correlation coefficient was Effort/Improvement (r = 0.919), followed by Important Role (r = 0.826) and Cooperative Learning (r = 0.806). In Ego Climate, the strongest association was found in Punishment for Mistakes (r = 0.809), followed by Unequal Recognition (r = 0.774) and Member Rivalry (r = 0.516). With regard to Victimization, the indicator with the highest correlation was Overt Verbal Victimization (r = 0.938), followed by Relational Victimization (r = 0.878), and Overt Physical Victimization (r = 0.775).

Likewise, significant associations (*p* < 0.005) were found between Task Climate and Victimization—being positive and direct (r = 0.208)—and between Task Climate and Physical Activity—also being positive and direct (r = 0.294). Statistically significant differences were also found at a level of *p* < 0.005 between Ego Climate and Victimization, revealing a direct association (r = 0.396), with an average correlation strength. With regard to the relationship between Ego Climate and the use of video games, the differences were also statistically significant (r = 0.250).

The use of video games and the practice of physical activity revealed a negative and indirect relationship (r = 0.085; *p* = −0.027), the correlation being week. However, the relationship between the use of video games and victimization was positive and direct, but correlation was also week (r = 0.094; *p* = 0.020). Task Climate and the use of video games did not reveal any statistically significant associations in school boys. The same occurred in the relationship between Ego Climate and the practice of physical activity, and the relationship between victimization and the practice of physical activity.

**Figure 3:**
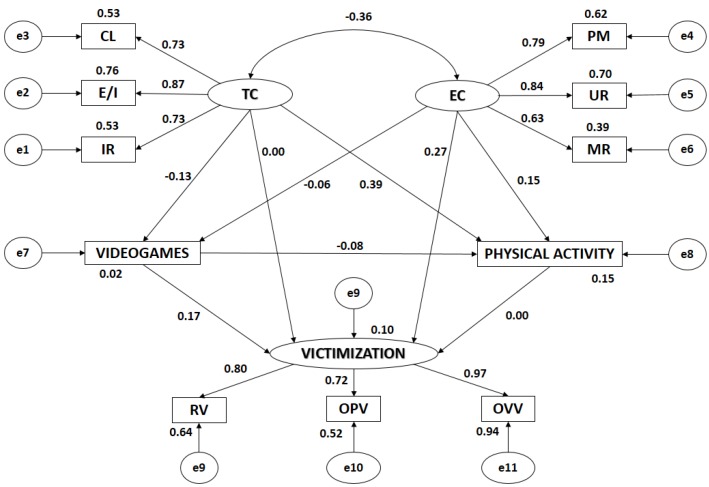
Structural equation model for girls. Note: TC, Task Climate; CL, Cooperative Learning; E/I, Effort/Improvement; IR, Important Role; EC, Ego Climate; MR, Member Rivalry; PM, Punishment for Mistakes; UR, Unequal Recognition; VIDEO GAMES, Use of video games; PA, Physical Activity; VICTIMIZATION, Victimization; RV, Relational Victimization; OPV, Overt Physical Victimization; OVV, Overt Verbal Victimization.

Figure 3 and Table 2 show estimated values of the parameters in the structural model for school girls. These must be of a suitable magnitude and the effects must be significantly different from zero. No improper estimations such as negative variances should be found.

Statistically significant relationships were found at the *p* < 0.005 level in all associations between motivational climate and its dimensions, all of these being positive and direct. The relationship between Task Climate and Ego Climate was significant at the *p* < 0.005 level, being negative and indirect (r = −0.360). Likewise, statistically significant relationships at the *p* < 0.005 level were found between victimization and its indicators, all of these being positive and direct.

Analyzing the influence of the indicators on each of the latent variables, statistically significant differences were found at the *p* < 0.005 level in all of them, all relationships being positive and direct. In Task Climate, the indicator with the highest correlation coefficient was Effort/Improvement (r = 0.871), followed by Important Role (r = 0.731) and Cooperative Learning (r = 0.728). In Ego Climate, the strongest association was found in Unequal Recognition (r = 0.839), followed by Punishment for Mistakes (r = 0.786), and Member Rivalry (r = 0.627). With regard to Victimization, the indicator showing the strongest correlation was Overt Verbal Victimization (r = 0.968), followed by Relational Victimization (r = 0.801), and Overt Physical Victimization (r = 0.722).

In the same way, significant associations (*p* < 0.005) were found between Ego Climate and Victimization -being positive and direct (r = 0.265)- and between Ego Climate and Physical Activity—being also positive and direct (r = 0.146), both having a weak correlation. Statistically significant differences were also found at the *p* < 0.005 level between Task Climate and the practice of physical activity, revealing a direct association (r = 0.393). With regard to the relationship between Task Climate and the use of video games, a negative and indirect association was found (r = −0.133), also having a weak correlation.

The use of video games and the practice of physical activity revealed a negative and indirect relationship (r = −0.081; *p* = 0.015), having a weak correlation. However, in the association between the use of video games and Victimization, the relationship was positive and direct, also having a weak correlation, although higher than the previous one (r = 0.174; *p* = 0.015).

Ego Climate and the use of video games did not reveal any statistically significant associations in school girls. The same occurred in the relationship between Task Climate and victimization, and between the practice of physical activity and Victimization.

## 4. Discussion

This research study conducted a multigroup structural equation analysis aimed at contrasting associations between the motivational climate in sport, victimization, the problematic use of video games, and the practice of physical activity. The path model developed had excellent fit indices and created a valid explanatory model enabling the understanding of the relationships that exist between motivational factors, victimization, the use of video games, and the practice of physical activity by schoolchildren, both boys and girls, in the same way as do other national and international studies [43,44,45,46].

Analyzing motivational climate, the proposed structural model reveals a significant and inverse relationship between task climate and ego climate for both boys and girls, which is stronger and more differentiated for girls. It seems evident that subjects strongly oriented towards the task show little motivation towards ego, and vice versa [47,48,49]. This is because individuals adopt a predominant orientation, either towards the task, which rewards effort and personal development, or towards ego, which fosters team member rivalry and the display of personal skill [50]. With regard to girls, this inverse relationship is stronger because boys are more ego-oriented and less task-oriented than girls, this being explained by the fact that girls tend to work towards intrinsic goals while boys focus on the achievement of extrinsic goals [51,52].

With regard to the categories that make up task climate dimensions, it can be seen that the influence of the indicators is proportional, with effort/improvement being the indicator with the strongest correlation, in both boys and girls, followed by important role and cooperative learning, the three indicators showing a stronger correlation in boys. In the case of ego climate, the most influential indicator for boys is punishment for mistakes, whereas for girls it is unequal recognition. These data are in line with the results of studies by Bandeira et al. [47] and Xiang et al. [53], which indicate that effort and improvement constitute the fundamental factor characterizing the type of motivation that is more self-determined or intrinsic, its focus being on improving and developing skills rather than thinking about external rewards. Since boys are more ego-oriented than girls, they are more concerned about punishment, whereas girls are influenced to a greater extent by unequal recognition, worrying less than boys about punishment and focusing more on intrinsic goals [49,51].

As for victimization, the influence of the indicators follows the same pattern in both boys and girls. For girls, however, correlation is slightly stronger in overt verbal victimization, while overt physical victimization and relational victimization have a stronger correlation for boys. The results indicate that girls suffer more verbal victimization than boys, while boys suffer more physical and relational victimization than girls [54,55]. In line with the findings of Devries et al. [56], it is clear that boys are more likely to victimize or be victimized physically, whereas in girls there tends to be a greater bias towards verbal aggression and victimization. The reason for this may be that boys are less fearful of physical aggression, as a result of social and cultural factors [57].

In terms of the relationship between motivational climate and victimization, a positive and direct relationship was found between task climate and victimization among boys, there being no such relationship in girls. This can be explained by the fact that task climate promotes cooperation, whereby individuals are obliged to work in a collective manner, interacting with others, which can easily give rise to incidents of bullying when the coach is absent or in unattended areas such as in hallways and changing rooms [58,59]. In terms of ego climate and victimization, a positive and direct relationship is found between them, for both boys and girls, but it is stronger for boys. The reason for this is that boys are more strongly oriented towards tasks than are girls. Therefore, when schoolchildren engage in physical activity, whether collectively or not, if that an activity is centered around competition, they may find themselves faced with defeat or disappointing results that cause them to experience frustration, which may lead to violent situations within the peer group [59,60].

A positive and direct relationship was found between the two motivational climates and the practice of physical activity, this being stronger in the case of task climate. Analyzed research indicates that the intention to be physically active is directly related to intrinsic motivation, which is connected to the more self-determined type of motivation, focusing on enjoyment and personal development [61,62]. The two types of motivational climate are not mutually exclusive; they are complementary and thus positively and directly related to the practice of sports. However, task climate is more beneficial in terms of commitment to participating in sports, ego climate being more oriented towards achieving results and showing off one’s skills [63,64].

Analyzing the connection between task climate and the problematic use of video games, a negative and indirect relationship was found for girls, showing an average strength of correlation, whereas this association is not significant for boys. In the case of ego climate and the problematic use of video games, they are directly and positively related for boys, but there is no such association for girls. Studies carried out by Gentile et al. [12] and Chacón et al. [65] show similar relationships, attributing them to the need for rivalry and the external reinforcement, provided by motivational climates oriented towards ego, this applying in virtually the same way to both sporting competitions and video games. In contrast, task climate can act as a protective factor against the pathological use of video games, since it promotes intrinsic motivation to engage in physical activity, increasing adherence to it and reducing sedentary use of digital entertainment [66,67].

The relationship between the problematic use of video games and the practice of physical activity is negative and indirect for boys and girls alike, its correlation being slightly stronger for boys. Coinciding with the results of research by Stubblefield et al. [68] and Krossbakken et al. [69], these findings are explainable by the fact that schoolchildren’s free time is usually devoted mainly to physical activity; however, since a couple of decades ago, this free time has been devoted increasingly to digital media such as video games. Therefore, these results indicate the need to encourage adherence to the practice of physical activity, to the detriment of sedentariness related to digital entertainment, in order to improve children’s health in general [70,71,72,73]. Additionally, boys tend to use video game consoles and video games more than girls, so their correlation is stronger [74].

A positive and direct relationship was found between the problematic use of video games and victimization in schoolchildren, the correlation being stronger in girls than boys. These data agree with other research studies consulted [75,76,77] in finding that young people who play video games with violent content tend to suffer more victimization than those who do not exhibit problematic use of video games [78,79,80]. Since most current video games do have violent content, constant exposure to them can affect children’s cognitive processes, leading to their perceiving violent behavior as normal and losing their sensitivity and empathy in the face of violent acts [23]. From this it can be deduced that the problematic use of video games becomes a risk factor in terms of school harassment or bullying and levels of sedentariness, negatively influencing both factors [81,82].

The present research study has some limitations, one of them being its cross-sectional nature, which precludes the establishment of cause and effect relationships. For such reasons, and in line with the data derived from the study, there is a need to implement intervention programs aimed at promoting adherence to the practice of physical activity instead of sedentary digital entertainment, in an attempt to replace time devoted to video games with healthy behavior related to the practice of physical activity and sport. It would also be interesting to promote motivational climates oriented towards task in the school context, in view of the inverse relationship that has been found with the problematic use of video games.

## 5. Conclusions

The findings reveal a positive relationship between task climate and the practice of physical activity for both genders, but it is stronger for girls. The association between task climate and the use of video games is negative and indirect for girls, whereas for boys it is not significant. Additionally, task climate and victimization is positively related for boys. Furthermore, ego climate and the use of video games are positively related for boys, but no association is found for girls. The relationship between the use of video games and the practice of physical activity is negative for both boys and girls, being stronger in the case of boys. The same tendency is followed between ego climate and victimization. Finally, there is a direct relationship between the use of video games and victimization for both genders, but the correlation is much stronger for girls. No relationship was found between victimization and the practice of physical activity, for either boys or girls.

## Figures and Tables

**Table 1:** Weights and standardized regression weights for boys.

Relationships between Variables			R.W.				S.R.W.
			Estimates	E.E.	C.R.	P	Estimates
VIDEO GAMES	←	TC	−0.561	0.430	−1.304	0.192	−0.056
VIDEO GAMES	←	EC	2.778	0.523	5.313	***	0.250
PA	←	TC	0.301	0.044	6.880	***	0.294
PA	←	EC	−0.039	0.053	−0.740	0.459	−0.034
PA	←	VIDEO GAMES	−0.009	0.004	−2.208	*	−0.085
VICTIMIZATION	←	TC	0.304	0.067	4.522	***	0.208
VICTIMIZATION	←	EC	0.644	0.083	7.760	***	0.396
VICTIMIZATION	←	PA	0.009	0.058	0.147	0.883	0.006
VICTIMIZATION	←	VIDEO GAMES	0.014	0.006	2.330	*	0.094
IR	←	TC	1.000	-	-	-	0.826
E/I	←	TC	1.139	0.043	26.325	***	0.919
CL	←	TC	0.963	0.041	23.662	***	0.806
PM	←	EC	1.000	-	-	-	0.809
UR	←	EC	0.467	0.031	15.203	***	0.774
MR	←	EC	0.726	0.062	11.730	***	0.516
OVV	←	VICTIMIZATION	1.000	-	-	-	0.938
OPV	←	VICTIMIZATION	0.679	0.027	25.495	***	0.775
RV	←	VICTIMIZATION	0.811	0.026	30.927	***	0.878
EC	↔	TC	−0.180	0.026	−6.815	***	−0.339

Note 1: TC, Task Climate; CL, Cooperative Learning; E/I, Effort/Improvement; IR, Important Role; EC, Ego Climate; MR, Member Rivalry; PM, Punishment for Mistakes; UR, Unequal Recognition; VIDEO GAMES, Use of video games; PA, Physical Activity; VICTIMIZATION, Victimization; RV, Relational Victimization; OPV, Overt Physical Victimization; OVV, Overt Verbal Victimization. **Note:** R.W., Regression Weights; S.R.W., Standardized Regression Weights; E.E., Error Estimation; C.R., Critical Ratio; *** Statistically significant relationship between variables at a level of 0.005; * Statistically significant relationship between variables at a level of 0.05.

**Table 2:** Weights and standardized regression weights for girls.

Relationships between Variables			R.W.				S.R.W.
			Estimates	E.E.	C.R.	P	Estimates
VIDEO GAMES	←	TC	−1.406	0.445	−3.159	***	−0.133
VIDEO GAMES	←	EC	−0.546	0.377	−1.448	0.148	−0.062
PA	←	TC	0.480	0.052	9.283	***	0.393
PA	←	EC	0.150	0.042	3.585	***	0.146
PA	←	VIDEO GAMES	−0.009	0.004	−2.422	*	−0.081
VICTIMIZATION	←	TC	0.000	0.078	0.004	0.997	0.000
VICTIMIZATION	←	EC	0.379	0.063	6.063	***	0.265
VICTIMIZATION	←	PA	0.005	0.053	0.103	0.918	0.004
VICTIMIZATION	←	VIDEO GAMES	0.028	0.006	4.939	***	0.174
IR	←	TC	1.000	-	-	-	0.731
E/I	←	TC	1.211	0.060	20.110	***	0.871
CL	←	TC	1.045	0.056	18.806	***	0.728
PM	←	EC	1.000	-	-	-	0.786
UR	←	EC	0.497	0.026	18.942	***	0.839
MR	←	EC	0.932	0.057	16.428	***	0.627
OVV	←	VICTIMIZATION	1.000	-	-	-	0.968
OPV	←	VICTIMIZATION	0.530	0.023	23.296	***	0.722
RV	←	VICTIMIZATION	0.790	0.030	26.585	***	0.801
EC	↔	TC	−0.173	0.023	−7.581	***	−0.360

Note: TC, Task Climate; CL, Cooperative Learning; E/I, Effort/Improvement; IR, Important Role; EC, Ego Climate; MR, Member Rivalry; PM, Punishment for Mistakes; UR, Unequal Recognition; VIDEO GAMES, Use of video games; PA, Physical Activity; VICTIMIZATION, Victimization; RV, Relational Victimization; OPV, Overt Physical Victimization; OVV, Overt Verbal Victimization; R.W., Regression Weights; S.R.W., Standardized Regression Weights; E.E., Error Estimation; C.R., Critical Ratio; *** Statistically significant relationship between variables at a level of 0.005; * Statistically significant relationship between variables at a level of 0.05.
